# Urban Biomining of Rare Earth Elements: Current Status and Future Opportunities

**DOI:** 10.1021/acsenvironau.5c00175

**Published:** 2025-11-22

**Authors:** Shuxin Zhang, Yun Shen

**Affiliations:** † Department of Civil and Environmental Engineering, School of Engineering and Applied Science, 8367 The George Washington University, 800 22nd Street NW, Washington, D.C. 20052, United States

**Keywords:** Rare Earth Elements, Electronic Waste, Bioleaching, Biorecovery, Recombinant Biomolecules, Microbial Platforms, Techno-Economic Analysis, Life Cycle Assessment

## Abstract

Rare earth elements (REEs) are critical to modern technologies and national security, playing essential roles in electronics, electric vehicles, and defense systems. Although they are not truly rare, their widespread but low-concentration presence in the Earth’s crust, combined with their chemical similarity, makes conventional mining both technically difficult and environmentally taxing. As a result, recycling REEs from electronic waste (e-waste), a practice often referred to as urban mining, has emerged as a promising alternative. However, current recycling methods face major challenges, including high energy demands, extensive use of harsh chemicals, generation of large volumes of solvent waste, and poor selectivity for REEs. These limitations significantly hinder the sustainability and scalability of REE recovery from e-waste, underscoring the urgent need for innovative, environmentally friendly strategies to extract and recover REEs. Recently, microorganism-based bioleaching and biosorption techniques have emerged as promising green alternatives to reduce the environmental burden caused by conventional recycling methods and further enhance the recovery efficiency and specificity of REEs from e-waste. Bioderived substances emerged as sustainable alternatives to upgrade the efficiency and specificity of REE exploitation and recovery from various resources. This review highlights three key areas essential for advancing REE biorecovery technologies, particularly in the context of urban biomining: (i) the use of bacteria-derived organic compounds as leaching agents for REE bioleaching from e-waste; (ii) the application of recombinant biomolecules, such as proteins, peptides, nucleic acids, and other engineered compounds, for selective biosorption and bioprecipitation of REEs; and (iii) the development and utilization of advanced microbial chassis and alternative nonchassis systems to enhance biorecovery efficiency. Key insights and future perspectives are provided to guide future design and advancement of integrated bioleaching–bioseparation systems for efficient and robust REE recovery from e-waste.

## Graphical abstract

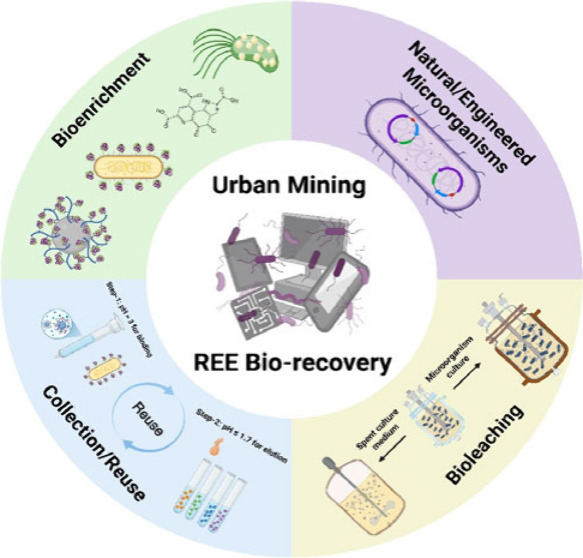

## 1 Introduction

Rare earth elements (REEs) are a group of 17 metallic elements, including those with atomic numbers 58 to 71 in the lanthanide series (lanthanum (La), cerium (Ce), praseodymium (Pr), neodymium (Nd), promethium (Pm), samarium (Sm), europium (Eu), gadolinium (Gd), terbium (Tb), dysprosium (Dy), holmium (Ho), erbium (Er), thulium (Tm), ytterbium (Yb), and lutetium (Lu)), as well as the transition metals scandium (Sc) and yttrium (Y). REEs, particularly Dy, Tb, Nd, and Pr, which are integral to rare-earth permanent magnets, are essential for a wide range of technologies, from high-tech consumer products like smartphones, laptops, and electric vehicles to critical defense systems such as lasers, radars, and guidance systems. Despite their relatively small quantities, REEs are crucial for the functionality of these products, making them indispensable for modern innovation, technological advancement, and even national security. The global demand for REEs is increasing rapidly and is projected to reach approximately 1.6 million tons annually by 2050.^1^ Although REEs are not particularly scarce in nature, they are seldom found in concentrated deposits, creating significant challenges for mining and extraction. Additionally, REEs tend to co-occur in natural deposits and share similar chemical properties, which complicates their purification and isolation. Conventional REE extraction from primary ores is highly energy-intensive and environmentally detrimental, driving interest in recovering REEs from secondary sources, such as waste streams.^2^ Recovering REEs from various waste streams, including mining tailings, coal ash, and electronic waste (e-waste), presents an alternative and promising source, helping to reduce dependence on primary mining and mitigating its negative environmental impacts.

With the boom of modern technology, electronic devices have largely facilitated and enriched people’s lives, while also raising concerns about e-waste. According to the World Health Organization’s report in October 2024, e-waste has become one of the fastest-growing solid wastes worldwide.^3^ The largest proportion of e-waste originates from household products (42.1%), followed by IT and telecommunications equipment (33.9%), consumer devices (13.7%), and small domestic appliances (4.7%).^4^ In 2022, among around 62 million tons of e-waste produced globally, less than one-quarter of the e-waste was documented to be recycled.^5^ Common e-waste, including smartphones, computers, household appliances, and medical devices, is usually recycled by unsound activities or stored at home, which can release various chemical substances, e.g., known neurotoxicants, lead, to the environment. These toxic substances pose potential risks to public health, particularly for vulnerable communities (e.g., pregnant women and children). Therefore, e-waste was classified as hazardous waste when cycled inappropriately. In contrast to its passive impact on public health, e-waste contains abundant critical metals, especially REE, that can be recycled and reintegrated into industrial production. This feature underpins the emergence of a growing field known as urban mining.^6^ The REE content varied among various types of electronic devices. For example, the permanent magnets in hard disk drives (Nd, Dy, and Pr) and the phosphors of fluorescent lamps (Y, Eu, Ce, Tb, and La) contain 2,500–15,000 ppm and 1,000–20,000 ppm of REE, respectively, which are the highest and are 17 times more than natural ores.^7^ The screens, speakers, and vibration motors of smartphones and the printed circuit boards, hard disk drives, and speakers of computers contain La, Ce, Pr, Nd, Sm, Eu, Gd, Tb, Dy, and Y with a concentration between 10 and 1,000 ppm.^8^ Moreover, according to the Global E-waste Monitor 2024 report by the United Nations Institute for Training and Research, e-waste is growing at a rate five times faster than documented recycling efforts.^9^ The abundant REE content and limited recycling infrastructure position e-waste as a promising and underutilized secondary source of REEs.^10^

Several nonbiological technologies have been reported to effectively enrich and separate REEs. For high-strength leachates from E-waste hydrometallurgy, membrane technologies, including nanofiltration and reverse osmosis, have shown high efficiency in concentrating REEs and reducing liquid volume.^11^ After coupling with diafiltration, nanofiltration can purify monovalent salts/acidity while retaining trivalent lanthanides, easing downstream polishing and reducing extractant consumption.^12^ With chelates or tailored cation-exchange membranes, electrodialysis has been reported to drive electrical enrichment and enable the differentiation between neighboring lanthanides.^13,14^ One recent experiment-validated modeling study successfully demonstrated Dy/Pr/Nd separation by using electrodialysis systems and provided design guidance for cost-effective scale-up.^15^ However, the membrane chemical stability under an acidic environment and fouling are key constraints for future practical implementation. Electrochemical pH-regulated hydrolysis showed the ability to separate aluminum from REEs through precipitation and can recover acid/base for reuse, offering reagent-saving alternatives to conventional hydrometallurgical processes.^16,17^ Nevertheless, tight pH control, corrosion control, acid/base recycle integration, and further processing of generated solids/sludge compromise its potential. For low-grade resources (e.g., coal fly ash leachates), REE contents are trace (50 to 300 μg/L), while competing ions (Ca^2+^/Fe^3+^/Al^3+^) are abundant, making high-affinity and highly selective ligands and adsorbents the preferred approach.^18,19^ Overall, while advanced nonbiological technologies deliver robust REE enrichment and group isolation, selectively recovering individual elements via greener and more sustainable pathways remains challenging.

Microorganism-based biorecovery of REE has emerged as a green and sustainable alternative to conventional hydrometallurgy due to its mild reaction conditions and low chemical and energy requirements, which involves two main processes, bioleaching and biosorption/bioaccumulation. Microorganisms, like *Acidithiobacillus*, heterotrophic bacteria (e.g., *Bacillus*, *Gluconobacter oxidans* (*G. oxidans*), and *Pseudomonas*), and some fungi, can metabolize metals through oxidation and dissolution, hence have been widely utilized as chassis/nonchassis leaching platforms for critical metal recovery.^20^ Based on whether there is direct physical contact between waste material and microbiomes, the bioleaching process could be classified into direct and indirect leaching (Figure 1a). In direct leaching (i.e., microorganisms directly mix and interact with ground wastes), metal mobilization is facilitated not only by strong oxidizing agents generated by microbial activity but also through enzymatically catalyzed metal dissolution occurring during microbial metabolism. Therefore, direct bioleaching has been reported to be more efficient than indirect bioleaching (i.e., wastes are mixed with cell-free cultured medium), although the latter is considered more suitable for industrial applications due to the absence of microorganisms, which allows greater flexibility in process optimization.^21,22^ In addition, previous studies also confirmed that, at a leaching condition of pH between 2 to 2.5, microorganism-based direct bioleaching of valuable metals (e.g., Cu, Ni, and Zn) is even more efficient than inorganic acids (e.g., HCl and H_2_SO_4_), and the maximum leaching could be achieved after 2 weeks of coincubation with mashed waste printed circuit boards (WPCBs).^23^ These findings strengthen confidence in the potential of microorganism-based bioleaching pathways as a viable alternative to conventional inorganic acid–based hydrometallurgy, which is often chemical and energy-intensive and waste-generating, supporting the development of low-carbon footprint and more sustainable methods for recovering critical metals from waste materials.

**Figure 1:**
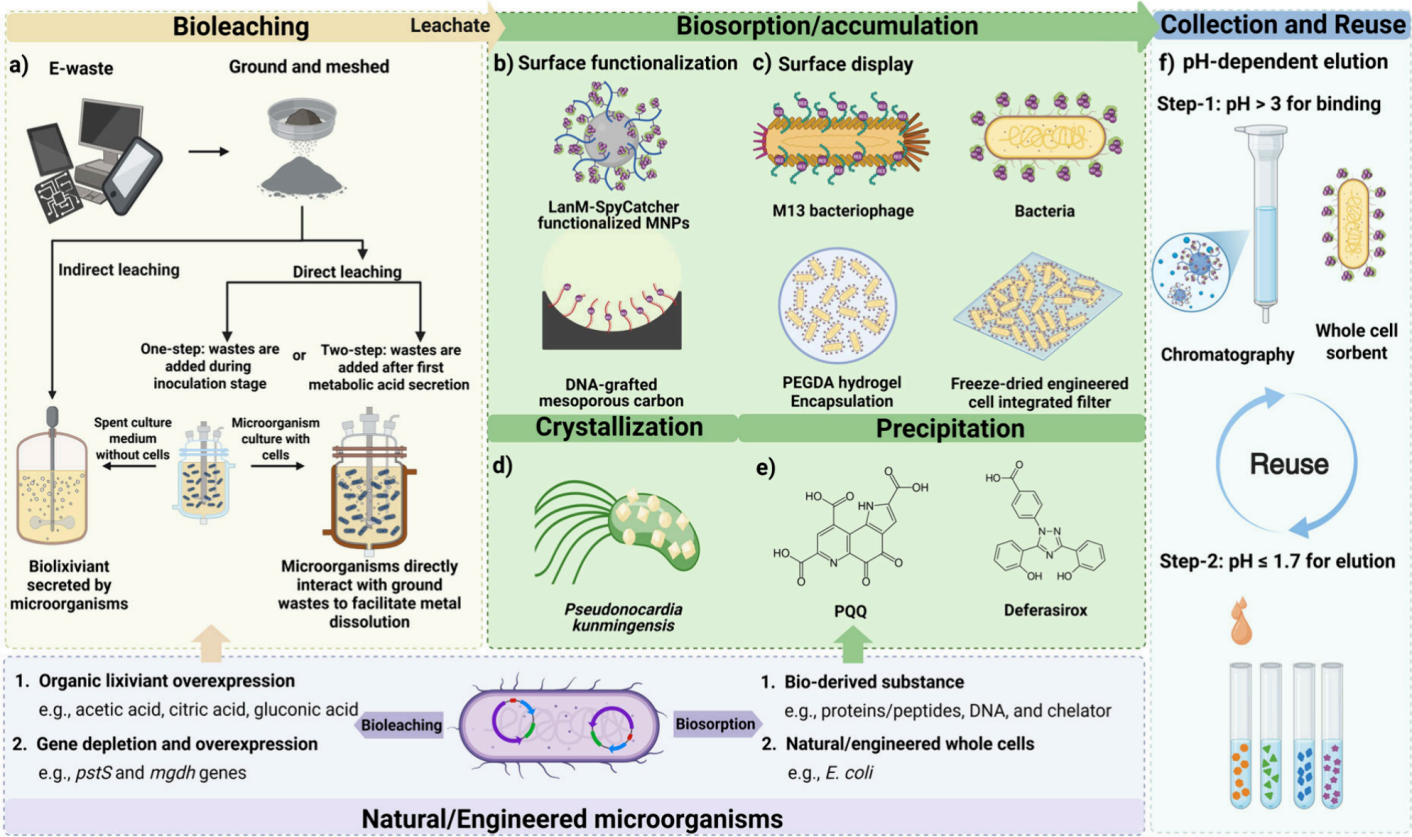
**Schemes of bioleaching and advanced strategies for REE biorecovery.** LanM: lanmodulin; MNPs: magnetic nanoparticles; PEGDA: Poly­(ethylene glycol) diacrylate; PQQ: pyrroloquinoline quinone.

Beyond bioleaching, microorganism-based biorecovery systems can also facilitate the intracellular accumulation and enrichment of REEs through metabolic processes. Except for bacteria, like *Pseudonocardia kunmingensis*, that have been reported to enable the *in situ* crystallization of REEs,^24^ natural REE-binding proteins, such as lanmodulin (LanM), lanpepsy (LanP), and pyrroloquinoline quinone (PQQ)-binding protein (PqqT), have been investigated and engineered for REE biosorption due to their high binding affinity and selectivity to REEs (Figure. [Fig fig1]b). Currently, LanM is regarded as the most promising candidate because of its stability under high temperature and acidic environment and its high selectivity for lanthanides over calcium, which has been engineered through multiple techniques to further enhance its REE binding performance. With the uncovering of structural binding mechanisms of LanM, a series of lanmodulin-derived peptides have been designed and engineered to elevate its application in selective REE biorecovery.^25,26^ Functional nucleic acids, especially aptamers, are also powerful tools that have been found to specifically bind REE ions in aqueous environments.^27^ Based on various binding kinetics, it has been reported that functional nucleic acids can discriminate REEs into three groups, which is promising to achieve the selective binding and the separation of even individual REEs.^28,29^ Besides, studies based on the cofactor or ligand of quinoproteins, like PQQ, revealed that PQQ can directly precipitate REEs from solutions at ambient conditions with a preference toward light REEs over heavy REEs^30^ (Figure 1e).

This review summarizes recent advances in microorganism-based strategies for REE recovery, covering both bioleaching and biosorption. Emphasis is placed on comparing the leaching efficiencies of various organic leaching agents, developing binding selectivity for individual REE separation, and establishing complete microbial platforms for REE recovery from e-waste. Emerging microbial-derived substances, such as natural and recombinant proteins, engineered peptides, functional nucleic acids, and novel chelators, are discussed to highlight their potential in enhancing the selectivity of REE bioleaching and biosorption. Additionally, the review covers potential microbial candidates and microorganisms engineered to produce organic lixiviants and REE-binding compounds, offering new components for establishing an integrated and efficient REE biorecovery system from e-waste. In the future, progress in synthetic biology and systems metabolic engineering will enable the development of modular, scalable microbial platforms designed for efficient, sustainable, and cost-effective urban mining of REEs.

## 2 Bioleaching of REEs from e-Waste

### 2.1 Chemical Composition of Various e-Wastes

Urban mining refers to the recovery of critical resources from waste and discarded manufactured goods generated in cities and industrial systems, rather than extracting them directly from natural ores and deposits. It is an emerging source recovery concept that refers to recovering valuable secondary raw materials from anthropogenic resources through biological, chemical, or physical processes based on technological innovation. By treating industrial byproducts and end-of-life materials as resources rather than waste, urban mining plays a key role in advancing the circular economy and reducing dependence on primary raw material extraction.^31^ E-waste is regarded as an emerging secondary resource for urban mining due to its concentrated composition of REEs and other precious metals. The main components in e-waste that are abundant in REEs include neodymium (NdFeB) magnets, WPCBs, and nickel metal-hydride (NiMH) batteries. Nd is the most common REE in NdFeB magnets and WPCBs, and it takes up to 30 wt % of the total chemical composition, while La is the main REE in NiMH batteries (16.2–23.2 wt %). Tb, Dy, and Gd are sometimes added into NdFeB magnets to replace Nd for increasing their operating temperature and intrinsic coercivity, while Pr, La, and Sm are added to reduce the production cost.^32,33^ Ce (2.7–8.5 wt %), Pr (0.07–7.10 wt %), Dy (0.43–6.3 wt %), Gd (0.02–1.51 wt %), Sm (0.77 wt %), and Y (0.042 wt %) are other REEs that are often found in e-waste. Studies estimate that REEs recycled from hard disk drives (HDDs) in the United States could supply up to 5.2% of the global demand (excluding China) for NdFeB magnets.^34^ Except for REEs, the e-waste also contains various base metals that compromise the REE bioleaching efficiency under certain conditions. For example, iron (Fe) is the main base metal of most e-waste with a proportion of 58.16–79.2 wt %, 7–38 wt %, and 0.02–15.4 wt % in NdFeB magnets, PCBs, and NiMH batteries, respectively. Copper (Cu, 10–27 wt %) and aluminum (Al, 2–19 wt %) take the other one-third total composition of PCBs, while Nickel (Ni, 41.6–46.6 wt %) is the main base metal of NiMH batteries.^33,35–37^ One study has reported that direct bioleaching by microorganisms can induce higher leaching efficiency of base metals, which might not be suitable for selective REE leaching and isolation compared to using spent culture medium.^38^ Given the diverse compositions of REEs and high concentrations of base metals in different types of e-waste, future bioleaching studies should focus on selective REE leaching to streamline and enhance subsequent isolation processes of individual REEs.

### 2.2 Organic Leaching Agents for e-Waste Bioleaching

Current bioleaching strategies often rely on organic leaching agents (e.g., organic acids, amino acids, and siderophores) secreted by microorganisms and can be enhanced by selecting optimal natural strains (e.g., bacteria like *Bacillus* and *Pseudomonas*, and fungi like *Penicillium* and *Aspergillus*) and leaching conditions (e.g., nutrient source and concentration, initial pH, and temperature), or by engineering microbial chassis (e.g., *E. coli*, *Bacillus subtilis*, *G. oxidans*) to overproduce suitable lixiviants.^39^ Table 1 lists the leaching conditions and performance of various leaching agents and microbial platforms for e-waste treatment.

**Table 1:** Leaching Performance of Various Leaching Agents and Microbial Platforms for e-Waste Treatment

e-waste	REE resources	Organic leaching agent	Conditions	Leaching performance	Reference
NdFeB magnets	NdFeB permanent magnets from end-of-life computer hard disk drives (HDDs)	Acetic acid, formic acid, citric acid, and tartaric acid	Acid concentration (10 vol % and up to saturation), and the solid/liquid (S/L) ratio (0.5–10%).	• Acetic acid demonstrates the highest REE leaching efficiency, achieving yields exceeding 90% for Nd, Dy, and Pr at acid concentrations of 1.6–10 mol/L and a solid-to-liquid ratio of 0.5–5% at 60 °C.	^40^
	NdFeB magnets from obsolete or defective mobile phones	Acetic acid and citric acid	Acid concentrations: 0.25, 0.50, and 1.0 M;	• Microwave-assisted leaching was the most effective method;	^42^
			S/L ratios: 1:100, 1:50, and 1:10;	• With microwaves, 0.5 M citric acid (S/L: 1/100) leached 57% of Nd and 58% of Pr, and 0.5 M acetic acid (S/L: 1/100) leached 48% of Nd and 65% of Pr, in 15 min.	
			Leaching durations: various time intervals;		
			Leaching techniques: microwave-assisted, ultrasound-assisted, and conventional leaching.		
	NdFeB permanent magnet scraps	Guanidine hydrochloride (GUC)-lactic acid (LA) deep eutectic solvents (DES), choline chloride (CC)-lactic acid (LA) DES, and ethylene glycol (EG)-maleic acid (MA) DES	Solid sample was introduced at a 1:100 S/L (w/w) ratio to the DESs and leached at 70 °C for 5 h.	• The REE leaching efficiency of GUC-LA DES was 22.5%; EG-MA DES got the highest selectivity, with a leaching efficiency of 97.3% Nd and 0.8% Fe;	^45^,^48^
				• The solvent could be reused at least twice, and the leaching efficiency of 97% for Nd and 0.7% for Fe was maintained.	
	Hydrogen decrepitated NdFeB powder	Citric acid and acetic acid	Solid-to-liquid ratio of 0.5 g magnet powder per 25 mL of acid; Operated at 23 ± 1 °C with constant stirring at 400 rpm; Leaching durations at 100, 200, 300 min, and 24 h; Initial organic acid concentrations of 0.1, 0.2, 0.4, 0.8, and 1 mol/L.	• The highest leaching efficiencies were achieved with 1 mol/L citric acid for Nd, Pr, and Dy (where almost 100% of the REEs were leached after 24 h) and 1 mol/L acetic acid (where >95% of the REEs were leached);	^41^
				• Fe and Co were coleached into the solution, and no leaching selectivity was achieved between the impurities and the REEs.	
Nickel metal-hydride (NiMH) batteries	Spent NiMH battery powder	d-gluconic acid sodium salt, 2-keto-d-gluconic acid hemicalcium salt, and 5-keto-d-gluconic acid potassium salt	1% (w/v) spent NiMH battery powder in 20 mL of solutions, Incubated at 27 ± 1 °C and 150 rpm for 14 days; a total salt concentration of 60 mM, with both individual and combined salts; pH conditions: uncontrolled, 3.0, 6.0, and 9.0, maintained by adjusting with nitric acid or sodium hydroxide every 2–4 days.	• Gluconate at a target pH of 3.0 ± 0.1 resulted in the highest overall leaching of REEs and base metals;	^50^
				• The average total REE leaching yields at pH levels of 9.0 ± 0.1, 6.0 ± 0.1, and 3.0 ± 0.1 were 7.8%, 11.5%, and 56.1%, respectively.	
	Manually disassembled, air-dried, ground, and sieved NiMH powder with a particle size below 100 μm	Siderophores produced by *Pseudomonas sp*.	0.1 g of purified siderophore was dissolved in 10 mL of deionized water and mixed with 0.1 g of NiMH battery powder, then incubated at 28 °C and 150 rpm for 8 days.	• The purified siderophore leached 14.8% of La, 3.9% of Nd, and 1.1% of Pr from the anode of a NiMH battery.	^51^
	NiMH battery powder that has been homogenized and screened through a 630 μm sieve	Gluconic and pyruvic acid produced by *G. oxidans* (DSM 3503) and *Streptomyces pilosus* (DSM 40097)	Various phosphorus sources were used for one-step, two-step, and spent culture medium bioleaching at 27 °C with 1% (v/v) bacterial inoculum, mixing at 150 rpm for 14 days.	• Greater REE leaching was achieved using *G. oxidans* spent-medium bioleaching (9.0% vs 6.0% total REEs), whereas two-step direct bioleaching resulted in higher base metal recovery.	^38^
Waste printed circuit boards (WPCBs)	Ground and sieved WPCB powder with a particle size of less than 75 μm	Organic acids produced by *Bacillus megaterium*; Gluconic acid was dominant in the presence of other acids such as citric, oxalic, succinic, lactic, tartaric, and malic acids	10 g/L pulp density, 60 °C, 160 rpm, and 24 h	• REE extraction efficiencies varied across different media, with Ce leaching reaching 30.3%, 23%, and 18%, while Dy leaching was observed at 6.9% and 6.5%, depending on the medium.	^52^

Organic acids, including acetic acid, citric acid, gluconic acid, formic acid, and tartaric acid, are common functional lixiviants generated during microbiome cultivation, which enable the solubilization of REEs and other valuable metals. These organic acids are often found as mixtures with various combinations and have been reported to facilitate multiple leaching preferences. For REE leaching of NdFeB powder, efficiency comparisons are mainly carried out for citric and acetic acids. Sahar et al. compared the leaching efficiency of acetic, formic, citric, and tartaric acids on NdFeB powder recovered from computer HDDs, and found that acetic acid (CH_3_COOH) exhibited the highest performance, achieving leaching yields exceeding 90% for Nd, Dy, and Pr with an acid concentration of 1.6–10 mol/L and a solid-to-liquid (S/L) ratio of 0.5%–5% at 60 °C.^40^ Marino et al. evaluated the leaching efficiency of citric and acetic acids on hydrogen-decrepitated NdFeB powder at 23 ± 1 °C, using an S/L ratio of 0.5 g of magnet powder per 25 mL of acid. After 24 h, 1 mol/L of citric acid achieved complete REE leaching (100%), slightly outperforming 1 mol/L of acetic acid (>95%). However, when combined with di­(2-ethylhexyl)­phosphoric acid (D2EHPA) in kerosene (Solvent 70), the acetic acid leachate exhibited significantly higher selectivity for Nd over Fe and improved extraction performance.^41^ Ronei et al. evaluated the leaching efficiency of acetic and citric acids on REE leaching from NdFeB magnets in obsolete or defective mobile phones with the assistance of microwave (175 °C) or ultrasound (without heating).^42^ Their results proved that microwave-assisted leaching is the most effective method compared to ultrasound and conventional techniques. Under microwave conditions, 0.5 M of citric acid (S/L 1:100) leached 57% of Nd and 58% of Pr, while 0.5 M acetic acid (S/L 1:100) achieved a leaching yield of 48% for Nd and 65% for Pr within 15 min. Although organic acids are able to leach overall REEs and have a comparable leaching performance to inorganic acids, studies confirmed that the spent culture medium that contains these organic acids yields even higher leaching efficiency.^43^ This may be attributed to the higher concentration and diversity of leaching lixiviants in the spent medium (i.e., cell-free medium after culturing), as well as the absence of direct contact between microorganisms and e-waste, which could be toxic and inhibit microbial metabolism.^22,38^

Natural deep eutectic solvents (NADESs) are mixtures of hydrogen bond acceptors and naturally derived carboxylic acids, sugars, and amino acids, which are stable, nonflammable, and biodegradable, and are regarded as green solvents. These chemicals can form eutectic mixtures with a melting point significantly lower than that of their individual components through strong hydrogen bonding. In REE leaching, deep eutectic solvents (DESs) can complex with REE ions and enhance their solubility via proton donation or ligand exchange, providing tunable selectivity, lower toxicity, and better biodegradability than traditional acidic lixiviants.^44^ DES often favors REE dissolution over base metals because their hydrogen-bond donors, typically carboxylic/sulfonic acids, provide hard O-donor chelation that matches the hard-Lewis-acid character of Ln^3+^, while chloride-based hydrogen-bond acceptors (e.g., choline or tetraalkylammonium chloride) stabilize REE chloro/oxo-species and facilitate ligand exchange more effectively than for Fe/Ni/Co.^45,46^ Spectroscopic work revealed lanthanide speciation in choline-chloride DESs depends strongly on the hydrogen-bond donor (HBD) and chloride coordination, so switching HBDs (or water content/temperature) can shift relative solubilities and, in some systems, enhance mid/HREE dissolution.^47^ Liu et al. and Seojin et al. investigated the leaching efficiency and selectivity of various organic acids-involved DESs and revealed that ethylene glycol (EG)-maleic acid (MA) DES can selectively leach Nd from NdFeB permanent magnet scraps, achieving a leaching efficiency of 97.3% for Nd while only 0.8% for Fe.^45,48^ Similarly, a tetraethylammonium chloride–levulinic acid DES selectively leached Nd from NdFeB, achieving ∼ 97.6% Nd dissolution with <0.44% Fe coleached (separation factor >9000).^49^ In addition to organic acids, other organic lixiviants, such as gluconate and its keto-derivatives, and siderophores, are emerging as promising reagents for achieving highly efficient and selective leaching of REEs over base metals. One study based on gluconate and its keto-derivatives for NiMH batteries leaching indicated that better REE leaching was observed under acidic conditions with gluconate (pH 3.0 ± 0.1), while more base metals were leached by 5-ketogluconate at pH ≥ 6.0.^50^ Purified siderophore from *Pseudomonas* sp. strain ASA235 showed the capability to leach approximately 14.8% of La from the anode of a NiMH battery, along with a smaller amount of Nd and Pr.^51^

In conclusion, acetic and citric acids are two main organic acids that can completely leach REE from e-waste under optimal conditions, and their leaching efficiency can be further improved by incorporating assistant techniques and substances. However, considering the downstream selective extraction and recovery of REEs, organic acids-involved DESs and other organic lixiviants could be optimized to facilitate selective leaching between REEs and base metals to improve REE purity. Furthermore, direct two-step bioleaching usually yields the highest leaching efficiency toward all metals (i.e., waste material is added after the bacteria culture achieves optimal metabolic activity and has accumulated sufficient bioleaching agents, rather than adding solid waste at the beginning of cultivation (i.e., direct one-step bioleaching)), which might impair the selective leaching of REEs. Therefore, the culture medium of a suitable natural/engineered microbiome cultured under optimal conditions might be the ideal candidate for REE leaching from e-waste.

## 3 Organic REE Binding Substances

REEs in aqueous solutions are usually trivalent cations, and they can bind to other substances mainly through three mechanisms, including electrostatic interaction, coordination, and ion exchange.^53^ The functional groups that contribute to interactions include −COOH, -MO (metal–oxygen bond), −SO_3_^–^, −OH, -NH_2_, and -NH-, enabling the adsorption of REEs through various bioderived substances.^54^ Table 2 classifies various REE-binding biosubstances along with their advanced applications, while Figure 2 illustrates their biological and chemical structures.

**Figure 2:**
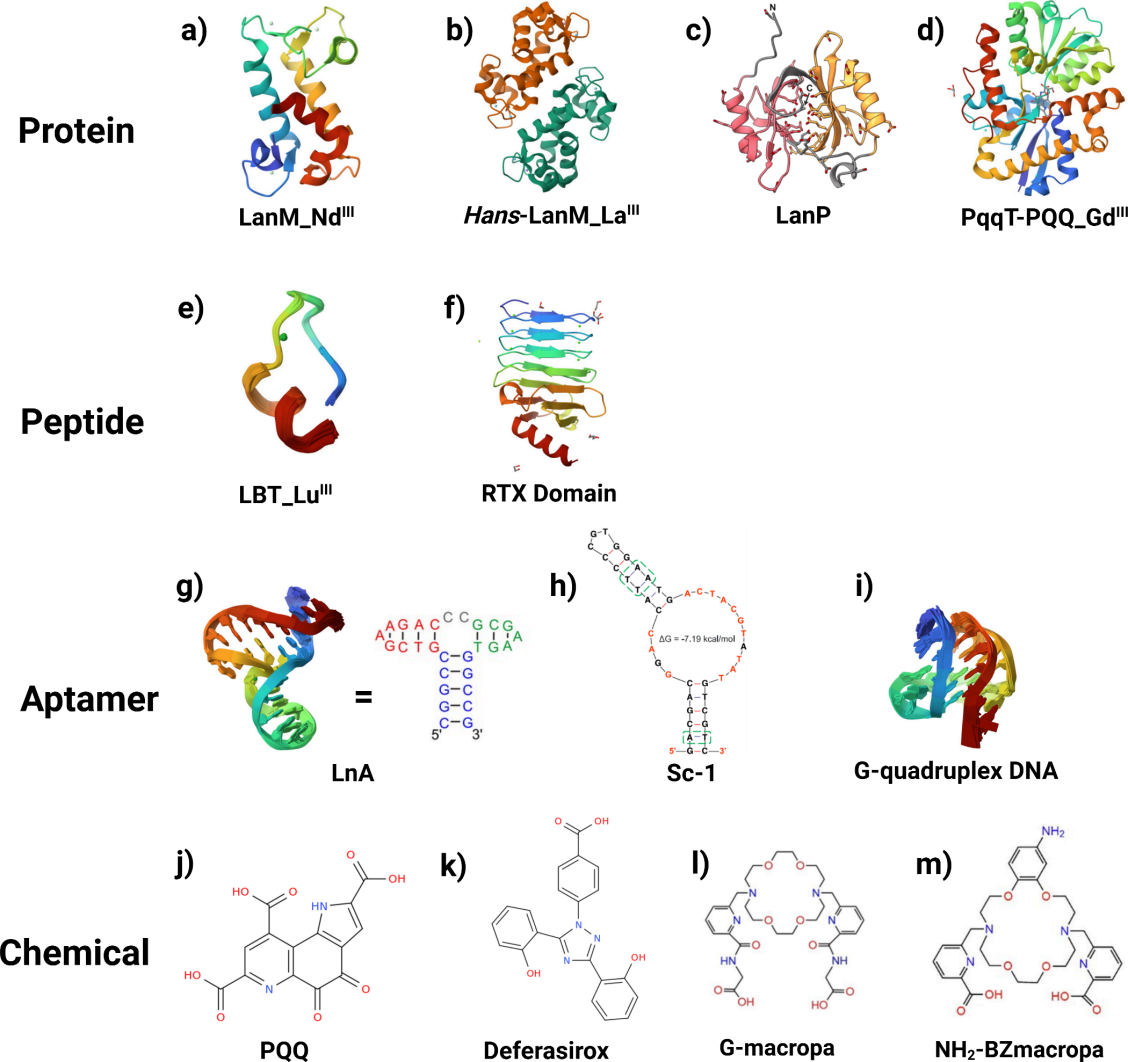
REE-binding substance structures. Protein: a) X-ray crystal structure of *Methylorubrum extorquens* AM1 LanM with Nd­(III) bound at pH 7 (PDB_8FNS); b) X-ray crystal structure of *Hansschlegelia quercus* LanM with Dy­(III) bound at pH 7 (PDB_8FNR); c) LanP monomer structure predicted by AlphaFold (Reproduced from ref 55. Copyright 2023 Elsevier); and d) Crystal structure of PqqT with PQQ and Gd­(III) bound (PDB_9B1 V); Peptide: (e) The binding structure of a lanthanide binding tag (LBT3) with Lu­(III) (PDB_7CCN); and (f) Crystal structure of the RTX region block V of *Bordetella pertussis* adenylate cyclase toxin; Aptamer: g) Solution structure (PDB_7QB3) and sequence of a lanthanide-binding DNA aptamer (Reproduced from ref 27. Available under a CC BY license. Copyright 2022 Witold. Andrałojć et al.); h) The Sc-1 aptamer structure predicted by mFold at 25 °C in 100 mM Na^+^ and 2 mM Mg^2+^ (Reproduced from ref 28. Copyright 2025 American Chemical Society); and (i) Human telomere DNA quadruplex structure in K^+^ solution hybrid-1 form (PDB_2HY9); and Chemical: j) Chemical structure of PQQ (CHEBI:18315); k) Chemical structure of deferasirox (CHEBI:49005); l) Chemical structure of G-macropa (Reproduced from ref 56. Copyright 2024 John Wiley and Sons); and m) Chemical structure of NH_2_–BZmacropa (Reproduced from ref 57. Copyright 2025 Springer Nature).

**Table 2:** REE-Binding Biosubstances

Substances and characteristics		Advanced applications	Binding performance	Reference
Proteins/Peptides	Lanmodulin (LanM):	Peptide-functionalized membrane adsorber by incorporating the hydrophilic monomer 2-hydroxyethyl methacrylate into a hydrophobic allyl methacrylate matrix	• Enhanced REE binding capacity in aqueous solutions was achieved;	^25^
	• ∼ 12 kDa and preferring to bind LREEs over HREEs;		• Despite a reduction in Nd/La selectivity (from 2.3 to 1.1), the copolymer design offered improved peptide immobilization and overall REE affinity;	
	• Binding affinity is very high (often pM-nM);		• REE binding was reversible and could be triggered by pH or competing ligands.	
	• Four metal-binding carboxylate-rich EF-hand motifs;	Lanmodulin-doped zinc imidazolate framework-8	• LanM improved MOF performance, leading to enhanced selectivity and adsorption capacity;	^62^
	• Very strong Ln^3+^ ≫ Ca^2+^/Mg^2+^ discrimination (often ≥ 10^6^ over Ca^2+^ in pH-relevant buffers);		• LanM@ZIF-8 exhibited a maximum adsorption capacity of 787.93 mg/g at 25 °C, significantly outperforming unmodified ZIF-8;	
	• Robust under pH of 2.5 and 95 °C.		• The material achieved adsorption equilibrium within just 1 h, markedly faster than conventional MOFs.	
		Lanmodulin-functionalized magnetic nanoparticles	• High adsorption capacity (6.01 ± 0.11 μmol-terbium/g), rapid kinetics, and >90% desorption efficiency was achieved;	^60^
			• Strong selectivity for REEs over non-REE metals was achieved, and the system exhibited enhanced protein stability;	
			• The system was magnetically recoverable and maintained ∼ 95% of its initial activity after eight reuse cycles;	
			• The system effectively enriched REEs from coal fly ash and geothermal brine leachates, achieving a 967-fold increase in REE purity.	
		Surface display of *E. coli* and freeze-dried cells as a sorbent	• Over 80% recovery of REE ions (Y^3+^, La^3+^, Gd^3+^, Tb^3+^), even in the presence of 100-fold excess competing ions, was achieved;	^77^
			• By incorporating into a filter, the system achieved a high capture capacity (12 mg/g dry cell weight), stability over ten reuse cycles, and week-long storage;	
			• A rapid, 5-min colorimetric assay enabled timely monitoring of REE recovery.	
	Lanpepsy (LanP):	It has not been applied to advanced material synthesis and engineering microbial chassis.	-	^55^
	• A periplasmic 19 kDa protein;			
	• 4–6 binding sites of REEs;			
	• Higher affinity of Ln^3+^ (*K*_d_ = 1 μM) than Ca^2+^ (*K*_d_ = 14 ± 11 μM).			
	Biomimetic rare earth artificial metalloprotein (PQQ ⊂ K142D-PqqT):	Mutation of the amino acid lysine (K_142_) to aspartic acid (D_142_) in PqqT.	• The addition of benzyl alcohol to La^3+^-bound PQQ⊂K_142_D-PqqT induced spectroscopic changes, indicating PQQ reduction;	^78^,^79^
	• A ∼ 33 kDa periplasmic protein;		• Chemical trapping confirmed the formation of benzaldehyde, supporting the alcohol dehydrogenase activity of the complex.	
	• One metal binding sits;			
	• Selective binding of La^3+^ (*K*_d_ = 0.6 ± 0.2 μM) over Ca^2+^ (*K*_d_ = 150 ± 30 μM).			
	Lanthanide binding peptides (LBPs):	LBPs-immobilized 4-methylbenzhydrylamine resin LL	• High adsorption efficiency (>80%) was achieved for Eu^3+^ and Tb^3+^;	^26^,^80^,^81^
	• Tb^3+^-binding peptide1 (ACVDWNNDGWYEGDECA) and Eu^3+^-binding peptide 2 (DPDK DGTIDLKE)		• Minimal adsorption was realized toward competing REEs with a similar ionic radius and valency;	
			• The system was reusable and maintained ∼ 60% of its initial adsorption capacity after five cycles.	
	Lanthanide-binding phages (LBPhs) based on LanM: Presenting ∼ 3300 copies of the peptide LanM	The major coat protein of M13 bacteriophage engineered with an LBP	• High REE binding capacity (35 mg/g, REE/phage) was achieved;	^66^
			• Preferential binding for heavy REEs over light REEs and minimal interaction with non-REEs was realized;	
			• REEs were released through pH modulation;	
			• Consistent ability to adsorb REEs over five cycles was achieved.	
	Lanthanide binding tags (LBTs):	*E. coli* engineered to display LBTs on the cell surface was encapsulated within a permeable polyethylene glycol diacrylate (PEGDA) hydrogel at high cell density	• Uniform cell distribution with accessible surface functional groups was prepared, enabling selective REE adsorption;	^72^,^73^
	• A greater than 60-fold affinity difference between REEs with the highest and lowest atomic number		• Nd extraction was effective in fixed-bed columns at flow velocities up to 3 m/h within pH 4–6;	
			• The system maintained 85% of adsorption capacity after nine cycles;	
			• Bench-scale testing with NdFeB magnet leachate showed a two-bed volume delay in REE breakthrough compared to non-REEs and achieved 97% REE purity in the adsorbed fraction at the breakthrough point.	
	Genetically encoded elastin-like polypeptides (RELPs):	A genetically encoded fusion of an elastin-like polypeptide (ELP) with the REE-binding domain of LanM	• The system selectively recovered high-purity REEs from simulated solutions containing trace concentrations (0.0001–0.005 mol %), achieving up to a 100,000-fold increase in REE purity;	^67^
	• Elastin-like polypeptide (ELP) and RELP of about 63 and 75 kDa;		• The system retained ∼ 95% of initial REE binding capacity after four cycles.	
	• Thermo (4 and 37 °C) and pH-responsive reversible phase-transition.			
	Repeat-in-toxin (RTX) domain:	*A. ferrooxidans* was genetically engineered to express LanM intracellularly and an RTX domain, periplasmically via fusion with the endogenous rusticyanin protein	• Both engineered cell lines showed enhanced recovery and selectivity for Tb^3+^, Pr^3+^, Nd^3+^, and La^3+^ over Fe^2+^ and Co^2+^ in synthetic magnet leachate;	^20^,^82^
	• A molecular size of 33.7 kDa for RTX fused with rusticyanin;		• The binding of Tb^3+^, Pr^3+^, Nd^3+^, and La^3+^ was improved by up to 4-fold for cells expressing LanM and 13-fold for cells expressing the RTX domains in both pure and mixed metal solutions;	
	• Binding about 8.7 equiv of La^3+^ ions.		• The presence of lanthanides in the growth media enhanced protein expression, likely by stabilizing the protein structure.	
Nucleic acid	Lanthanide-binding aptamers (LnAs): A 44-nucleotide Ln-aptamer	Adapted into a fluorogenic sensor for detecting Gd^3+^ in aqueous solutions	• The low detection limit of ∼ 80 nM helped ensure a high level of purity;	^27^,^83^
			• The sensor was relatively unresponsive toward many other metal ions;	
			• Some cross-reactivity was observed with other trivalent lanthanide ions, including Er^3+^ and Tb^3+^.	
	Sc-1 aptamer:	Combined with thioflavin T fluorescence assay and EDTA to specifically detect Sc^3+^, distinguishing LREEs, HREEs, and non-REE ions	• Favorable binding of to HREEs over LREEs was realized;	^28^
	• A 42-nucleotide sequence with secondary structures;		• Sc-1 exhibited distinct binding kinetics with trivalent lanthanide ions, enabling the classification of 17 REEs into three groups: (1) La^3+^, Ce^3+^, Pr^3+^, Nd^3+^, Sm^3+^, Eu^3+^, and Gd^3+^; (2) Tb^3+^, Dy^3+^, Ho^3+^, Er^3+^, Tm^3+^, Yb^3+^, Lu^3+^, and Y^3+^; and (3) Sc^3+^;	
	• Only binds to REEs, but not other metal ions.		• *K*_d_ of Sc-1 ranged from 0.6 to 258.5 nM for the REE ions.	
	Single-strand DNA: Three types of single-stranded DNA were used, one with 100 thymine bases, another with 20 thymine bases, and the third consisting of 2000-base-long DNA extracted from salmon milt	DNA-functionalized mesoporous carbons featured a BET surface area of 605 m^2^/g and a median mesopore diameter of 48 Å	• All DNA-functionalized mesoporous carbons showed higher REE adsorption than pristine mesoporous carbon, where the variant grafted with 100 thymine units achieved slightly better adsorption performance than others;	^84^
			• The system achieved an adsorption capacity of 110.4 mg/g for Nd^3+^ at an initial concentration of 500 mg/L;	
			• REE recovery was feasible at lower pH conditions;	
			• Adsorption was more effective for lower concentrations of REEs.	
	G-quadruplex DNA structure:	-	• LREEs replaced Na^+^ or K^+^ in G-quadruplexes and formed a more compact LREE-induced G-quadruplex structure;	^85^
	• The structure was formed by a planar arrangement of four guanine bases stabilized through Hoogsteen hydrogen bonding		• The thymine in the central loop of the human telomeric sequence contributed to the stabilization of G-quadruplex structures induced by LREEs.	
	• The structure was stabilized by cations in its central cavity;			
	• Binding stoichiometry of lanthanide ions to telomeric variants was 2:1.			
Chemicals	Pyrroloquinoline quinone (PQQ): A quinone and redox enzyme cofactor	Na_2_PQQ was used to precipitate lanthanides from aqueous solutions.	• PQQ instantly precipitated one equivalent of lanthanides and was fully recovered and separated from lanthanides by adding concentrated HCl;	^30^
			• PQQ preferentially formed complexes with early lanthanides, which rapidly precipitated from aqueous solutions;	
			• The observed separation was likely influenced by the ionic size of the lanthanides.	
	Deferasirox derivatives:	Competitive precipitation in the presence of triethylamine	• Chemicals and REEs formed 2:2 complexes in the solid state;	^86^
	ExPh (1), ExBT (2), ExCF_3_ (9), ExNMe_2_ (11), and ExSO_3_H (10)		• Under competitive precipitation conditions with triethylamine, high selectivity (up to 80%) for Lu(III) over La(III), Ce(III), and Eu(III) was achieved, with theoretical calculations supporting the selective crystallization behavior;	
			• The choice of base was critical for optimizing Lu(III) selectivity, with triethylamine yielding the highest selectivity.	
Whole microbes	• Four strains: *E. coli*, *Bacillus sphaericus*, *Bacillus mycoides*, and *Bacillus cereus*;	Four typical REE adsorption strains and the bacterial structural components were compared for REEs and non-REEs (Mn and Zn) adsorption.	• *E. coli* effectively enriched REEs, outperforming non-REEs and leading to the fractionation of HREEs and LREEs;	^53^
	• Bacterial structural components: Freeze-dried powder, cell walls, extracellular polymeric substances, and intracellular components.		• Four cycles of *E. coli* for REE adsorption demonstrated the reusability of the microbes for REE recovery from mining wastewater;	
			• The recovery mechanisms for REEs involved electrostatic attraction and ion exchange.	

### 3.1 Proteins and Peptides

Advancements in REE biorecovery have led to the identification of numerous organic ligands with high specificity for binding REEs. Among them, natural proteins and their derived peptides have emerged as a research focus due to their high binding affinity and selectivity for REEs over other metals. Lanmodulin (*Mex*-LanM), identified as the first natural REE-binding protein, was discovered through a targeted proteomic analysis of low-molecular-weight proteins coeluted with the lanthanide-dependent methanol dehydrogenase XoxF from *Methylobacterium extorquens* (*M. extorquens*).^58^ LanM has a small molecular weight of ∼ 12 kDa and has a superior selectivity (10^6^-fold) of Ln^3+^ over Ca^2+^, enabling a picomolar binding affinity toward lanthanides, especially for light REEs (LREEs) like La^3+^, Nd^3+^, and Sm^3+^ (dissociation constants, *K*_d_ = ∼ 5 pM). Compared to base metals such as Cu^2+^ and Zn^2+^, LanM shows a 10^5^∼10^7^-fold higher affinity for Ln^3+^, enabling strong discrimination between rare-earth ions and these competing cations.^59^ In addition, it has been validated that *Mex*-LanM is robust to maintain structural stability under high temperature (up to 95 °C) and acidic environments (pH 2.5). Several LanM-functionalized materials, such as SpyTag-functionalized magnetic nanoparticles (MNPs),^60^ porous support materials,^61^ and ZIF-8,^62^ have demonstrated significantly enhanced sorption capacity and selectivity for REE recovery compared to their unmodified counterparts, validating the stability of LanM after immobilization (Figure 1 b). These results highlight the promise of using LanM for industrial-scale REE isolation and purification. In 2023, a new type of LanM, *Hans*-LanM, was isolated from *Hansschlegelia quercus*, which also prefers LREEs over heavy REEs (HREEs).^63,64^ *Hans*-LanM exhibits substantial sequence divergence from *Mex*-LanM, sharing only 33% of sequence identity, including differences in metal-binding sites. It enables efficient single-phase baseline separation of Dy^3+^ from Nd^3+^, achieving over 98% purity and more than 99% yield, demonstrating greater selectivity than *Mex*-LanM. LanM exhibits high selectivity for REEs due to several unique structural features. It contains four carboxylate-rich EF-hand motifs, each comprising 12 residues that form metal-binding loops flanked by alpha-helices, enabling strong coordination with REEs. Unlike typical Ca^2+^-responsive EF-hand proteins, which have 25-residue spacings between EF-hands, LanM features a more compact 12–13 residue spacing, resulting in an atypical triple-helix bundle structure with metal-binding sites exposed on the periphery. Additionally, the presence of proline residues in the second position of at least one EF-hand may hinder interactions with Ca^2+^ and other non-REE metals, contributing further to its exceptional metal selectivity. With the reveal of the LanM structure, multiple LanM-derived peptides were designed based on the EF-hand structure in LanM. These peptides have a molecular weight of ∼ 1.6 kDa, making them easier to design and select, while also allowing for a higher loading capacity in surface display applications. Their surface display stability and natural affinity for lanthanides in solutions, on membranes, and on gold sensors in solutions where pH 4 ∼ 6 have also been proved.^25,65^ Consequently, a growing number of computationally designed peptides have been developed and applied in the modification of resins,^26^ bacteriophage,^66^ and microorganisms,^20^ as well as in the synthesis of functional polypeptides,^67^ offering a promising avenue for creating novel binding biomaterials with high affinity and selectivity.

Lanthanide binding tags (LBTs) are genetically encodable tiny (∼12–20 amino acids) peptides originally engineered to study the structure, function, and dynamics of proteins, and can selectively bind Ln^3+^ with high affinity (*K*_d_ of nM-μM).^68^ Most LBTs are designed based on calcium-binding EF-hand loops and use carboxylate side chains (Asp/Glu) and backbone carbonyls to provide high-affinity, site-specific coordination.^69^ Some designs incorporate an aromatic “antenna” residue (e.g., Trp) that sensitizes Tb^3+^/Eu^3+^, allowing for bright, time-resolved luminescence.^70^ LBTs can be produced inexpensively through fermentation and then fused or multimerized to increase capacity. Since they are site-specific tags, they can be immobilized in an oriented way on the cell surface,^71^ and further encapsulated in hydrogels,^72^ which improves reproducibility and reduces ligand loss. Although LBTs show excellent discrimination of Ln^3+^ over Ca^2+^/Mg^2+^, their ability to distinguish across individual REEs and to differentiate REEs from other trivalent metal ions, e.g., Fe^3+^/Al^3+^, is modest.^73^ Compared with LanM, LBTs show reduced stability in acidic media, where protonation of carboxylate-rich binding loops diminishes metal binding and decreases apparent affinity.^74^ In addition, LBT performance also varies along with buffer composition because common buffers (HEPES (hydroxyethylpiperazine ethanesulfonic acid)/MOPS (3-(N-morpholino) propanesulfonic acid)/MES (2-(N-morpholino) ethanesulfonic acid)/PIPES (piperazine-N, N′-bis (2-ethanesulfonic acid)) can competitively bind Ln^3+^ and perturb speciation.^75^

LanP is a 19 kDa periplasmic protein recently found in the obligate methylotroph *Methylobacillus flagellates*.^55^ LanP is the first known member of the PepSY protein family capable of binding REEs, containing two typical PepSY domains and up to four high-affinity Ln^3+^ binding sites that are primarily coordinated by negatively charged glutamate and aspartate residues, which is similar to the well-known LanM protein, despite having little sequence or structural similarity. Isothermal titration calorimetry confirmed LanP’s ability to bind various lanthanides (e.g., Ce^3+^, La^3+^, Nd^3+^, Y^3+^, and Pr^3+^) and Ca^2+^, with binding affinities surpassing those of the standard dye Arsenazo III. Arsenazo III is used as a standard dye for metal titration due to its high sensitivity and selectivity for forming stable, intensely colored complexes with metal ions, especially rare earth and actinide ions. Its strong absorbance in the visible range allows accurate, low-concentration detection via simple spectrophotometric methods, making it ideal for quantitative metal analysis.^76^ The study results indicated that, although *M. flagellatus* lacks LanM, LanP may functionally substitute for LanM to enable the metal binding and transport *in vivo*. Notably, under tested conditions, LanP did not directly influence Ln^3+^ uptake, XoxF expression, or cell growth. While research on LanP is still in its early stages and its selectivity for Ln^3+^ (*K*_d_ = 1 μM) over Ca^2+^ (*K*_d_ = 14 ± 11 μM) is not comparable to LanM, its relatively smaller molecular size and high binding capacity (4 ∼ 6 binding sites) for REEs suggest significant potential for industrial-scale lanthanide separation.

### 3.2 Nucleic Acids

Nucleic acid–based materials have shown strong potential for capturing REEs due to their phosphate and oxygen functionalities. Inspired by the natural REE-binding properties of bacterial cell walls, which are rich in phosphate groups, researchers have utilized DNA as an effective adsorbent for a wide range of REEs.^87,88^ Further advancements include the development of DNA-cellulose hybrid materials for REE separation, demonstrating successful adsorption of Nd, Dy, and Lu.^89^ DNA-functionalized mesoporous carbon significantly enhances the REE adsorption and selectivity, particularly Nd­(III), with adsorption efficiency influenced by DNA types, pH, and REE atomic properties. The results were confirmed by X-ray absorption near edge structure (XANES) and extended X-ray absorption fine structure (EXAFS) analyses, reinforcing the applicability of nucleic acid in sustainable and selective REE recovery systems.^84^

Although DNA contains numerous metal-binding groups, it generally exhibits low affinity for metal ions. The typical *K*_*d*_ for metal binding by nucleotides ranges from the high micromolar to millimolar levels. Many catalytic DNAs (DNAzymes) can detect target metal ions at concentrations as low as the nanomolar or even picomolar range, but their apparent binding affinity typically remains in the low micromolar range. However, DNA has the potential to bind REE ions more strongly, as these ions can interact with both the phosphate backbone and nucleobases.^90^ Furthermore, the interactions between DNA and Ln^3+^ primarily involve phosphate groups in nucleotides like cytidine and thymidine monophosphate (CMP and TMP). While nucleobases in adenosine and guanosine monophosphate (AMP and GMP) also contribute, phosphate groups are essential for effective binding, as nucleosides alone cannot chelate Ln^3+^. This binding is largely entropy-driven, especially with phosphate interactions, and becomes more pronounced with an increasing atomic number of Ln^3+^. Notably, with GMP, the reaction shifts from exothermic to endothermic around Gd^3+^, marking the “gadolinium break” seen in many Ln^3+^-mediated RNA cleavage processes.^83^ This observation indicates a promising strategy for the selective binding of Ln^3+^ ions preceding Gd^3+^ through the controlled adjustment of ion concentration and temperature. Jin et al. isolated an aptamer named Sc-1 through systematic evolution of ligands by exponential enrichment (SELEX) to selectively bind REE ions, especially Sc^3+^, showing strong affinity (*K*_*d*_ = 0.6 ∼ 258.5 nM), resistance to EDTA dissociation, and binding-induced structural changes.^28^ Thioflavin T (ThT) fluorescence assays showed that Sc-1 binds REEs but not other metal ions. Its distinct kinetics with different REEs enabled classification into three groups and allowed effective detection of Sc^3+^ in real samples. Another aptamer, Tb-1, has been reported to bind exclusively to REEs, exhibiting strong and selective affinity for a broad range of lanthanide ions.^29^ Compared to Sc-1, Tb-1 displays faster exchange with EDTA, suggesting it functions as an outer-sphere ligand. Its high sensitivity enables detection of Tb^3+^ at concentrations as low as 0.5 nM in environmental samples, making it a valuable tool for REE sensing and separation from low-grade REE sources. In addition, G-quartets are planar structures formed by four guanines via Hoogsteen hydrogen bonding. These quartets stack to form G-quadruplexes, columnar 3D structures, stabilized by cations in their central cavity.^91^ Sampat et al. reported that micromolar concentrations of LREEs can induce G-quadruplex formation and promote the formation of unimolecular, with a 2:1 binding stoichiometry.^85^ LREE binding also induces conformational changes in preformed Na^+^- or K^+^-stabilized G-quadruplexes by displacing these monovalent cations, resulting in a more compact structure, and the thymine in the central loop contributes to stabilizing the LREE-induced G-quadruplex.

Overall, nucleic acid–based substances, such as aptamers, exhibit good binding affinity toward REEs and have the unique capability to discriminate among different REE groups, highlighting their significant potential for enabling selective separation and purification of individual elements. Although the expression of aptamers in microbial chassis for REE binding remains largely unexplored, recent advancements, such as the successful *in vivo* expression of circular RNA aptamers, provide a promising foundation.^92,93^ These developments open new avenues for incorporating engineered nucleic acids into microbial platforms for targeted and efficient REE biorecovery.

### 3.3 Other Substances

Biologically derived chelators and ligands are also promising candidates for facilitating the direct precipitation of REEs from aqueous environments by forming low-solubility REE-containing complexes. REE^3+^ ions are hard Lewis acids that preferentially bind hard Lewis bases (e.g., O and N donors), with high coordination numbers (typically 8–10). Common contributors include phenolic or catecholic oxygen atoms (−OH on aromatic rings, often deprotonated to – O^–^), carboxylate groups (−COO^–^), carbonyl oxygen (C = O, from amides, esters, and quinones), secondary or tertiary amine nitrogen (−NR_2_, especially when part of macrocycles), and macrocyclic frameworks that preorganize donor atoms to fit the REEs’ coordination geometry (Figure 2). For example, PQQ is a vital redox cofactor in bacterial calcium- and lanthanide-dependent methanol dehydrogenase (MDH). PQQ has a chemical structure that contains multiple carbonyl oxygens and pyridine-type nitrogen within a rigid quinone-pyrroloquinoline ring, forming an ONO “pincer” motif that provides ideal tridentate chelation for lanthanides (Figure 2j). *In vivo*, it can bind with high affinity (*K*_d_ = 50 nM) to one equivalent of the periplasmic binding protein PqqT, enabling the uptake of exogenous PQQ to supplement endogenous cofactor biosynthesis. Researchers used it then to form an artificial metalloprotein with La^3+^, i.e., La^3+^-bound PQQ ⊂ K_142_D-PqqT, which carries a K_142_D mutation, enabling the conversion of benzyl alcohol to benzaldehyde *in vitro*.^78,79^ This mutation alters the binding affinity for La^3+^ and Ca^2+^ from 6 ± 1 μM and 64 ± 5 μM to 0.6 ± 0.2 μM and 150 ± 30 μM, respectively, demonstrating a remarkable increase in selectivity for La^3+^ over Ca^2+^. This enhanced specificity highlights its potential as a selective REE sorbent. In addition, PQQ has been reported to rapidly and singly precipitate lanthanides from neutral aqueous solutions of its sodium salt (Na_2_PQQ) at room temperature, exhibiting a 1:1 stoichiometry even in the presence of excess lanthanides (6 equiv).^94^ The Ln-to-La ratios in the PQQ–Ln (PQQ/Ln or La = 1:1) complexes revealed a binding preference of PQQ for LREEs, with approximately 55% complexation observed for Ce–Eu, compared to less than 30% for HREEs (Ho–Lu).^30^ Deferasirox, an FDA-approved iron chelator for treating iron toxicity, has been shown in a previous study to form 2:2 complexes with lanthanides in the solid state through its derivatives, where metal ions coordinate with the phenolate oxygen and triazole nitrogen (Figure 2k). Under competitive precipitation conditions with triethylamine, its derivatives exhibited high selectivity (up to 80%) for Lu­(III) over La­(III), Ce­(III), and Eu­(III).^86^

G-macropa, a macropa analogue, was developed for aqueous precipitation-based separation of Nd^3+^ and Dy^3+^ due to the circular structure and functional groups.^56^ The REE^3+^ ion is encapsulated within the macrocyclic cavity, coordinated by four nitrogen donors from the ring backbone and multiple oxygen donors from the pendant acetate arms, with an inner-sphere water molecule occasionally present depending on the ionic radius of the metal. In the presence of bicarbonate, Dy^3+^ selectively precipitates as Dy_2_(CO_3_)_3_, while Nd^3+^ remains in solution as a G-macropa complex. This method achieved high separation factors (up to 841) in both model mixtures and real magnet waste. G-macropa was also efficiently recovered and reused via crystallization in HCl, demonstrating good recyclability. Besides, a bifunctional chelator, NH_2_–BZmacropa, has recently been studied for Ln^3+^ precipitation and was immobilized on resins for selective REE extraction and separation.^57^ It retains the same macrocyclic N, O-donor arrangement as G-macropa but is rigidified through the incorporation of aromatic linkers. Its amide-like nitrogen atoms and carboxylate groups coordinate the REEs, while the increased rigidity helps reduce the required entropy loss of ligand (entropic penalty) associated with binding. Considering the binding of PQQ and PqqT protein, although these chelators are not naturally synthesized by organisms and their REE precipitation usually requires a neutral environment (pH 7), they could be considered to modify other biomolecules, like proteins/peptides, to achieve selective precipitation in acidic solutions.

Moreover, microorganisms themselves could also be used for REE sorption. One study evaluated the sorption efficiency of four strains of bacteria and different bacterial cell components.^53^ The results indicated that *E. coli* yielded the highest REE recovery from acidic mining wastewater with a preference for HREEs, especially Yb and Lu, and demonstrated stability within four adsorption–desorption cycles. However, compared with *E. coli*, the adsorption by the three *Bacillus* strains was relatively low, and they had varying degrees of preference for La, Nd, and Y. These results underscore the significance of host cell selection in developing REE biorecovery platforms with high selectivity.

Among REE-binding biomolecules, LanM exhibits superior performance for REE recovery, combining high stability under acidic and elevated-temperature conditions with exceptional intrinsic selectivity for Ln^3+^ over base metals (∼10^6^-fold) and measurable discrimination among adjacent REEs. Recent work exploits pH and eluant programming and protein engineering to resolve Ln groups and bias An/Ln recognition for separations.^95,96^ DNA aptamers selected by capture-SELEX achieve low-nanomolar affinities that are largely specific to REEs (minimal response to nonlanthanide ions), with sequence-dependent trends that often favor mid-to-heavy Ln^3+^, enabling group-level readouts and potential pairing with downstream capture chemistries. Given their facile synthesis and SELEX-based evolvability, nucleic-acid aptamers are promising candidates for element-specific recognition of individual REEs. However, in strongly acidic media (pH ≤ 3), base protonation and acid-catalyzed backbone cleavage destabilize aptamer folds, reducing affinity and selectivity. Therefore, maintaining performance typically requires acid-stabilization strategies, e.g., embedding the aptamer in protective matrices or coligand scaffolds that buffer the local pH. In solution, PQQ shows a binding preference toward LREE over HREE across lanthanides, it can be biologically synthesized, and it can be cocomplexed with natural proteins, promising its future applications in REEs biosorption/precipitation. Likewise, G-macropa forms very stable complexes, typically favoring LREEs, providing strong discrimination against base metals and, in several cases, outperforming other chelators in separation applications.

## 4 Microorganism-Based REE Biorecovery Systems for e-Waste Treatment

Microorganism-based systems for recovering REEs from e-waste represent a promising research future with substantial environmental and economic benefits. Two main directions include the development of comprehensive microbial platforms that integrate bioleaching and biosorption/bioaccumulation functions, and the implementation of sequential bioleaching and biouptake processes using diverse natural/engineered microbial strains. A comprehensive understanding of the preferences, mechanisms, and key influencing factors underlying REE bioleaching and biosorption/bioaccumulation by various microbial strains lays a strong foundation for developing highly efficient and selective REE biorecovery systems tailored to e-waste treatment. Table 3 summarizes the performance, strengths, weaknesses, and engineering opportunities of several microorganisms for REE biorecovery.

**Table 3:** Advantages, Limitations, and Engineering Opportunities of Natural/Engineered Microorganisms for REE Biorecovery

Microorganisms	Performance in bioleaching/biosorption	Advantages	Limitations	Engineering opportunities
*Escherichia coli*	• Natural binding preference for Yb/Lu.	• Versatile chassis;	• Acid sensitivity limits applications for harsh leaching conditions.	• Add acid-tolerance traits;
		• Modular surface display;		• Express highly selective REE-binders;
		• Whole-cell biosorbent.		• Boost value-added organic-acid generation for bioleaching.
*Acidithiobacillus*	• Highly acidic (pH ∼1–2);	• Natural acid tolerance;	• Nonspecific: codissolution (Fe, etc.) can hinder REE recovery.	• Pretreat to remove Fe and other impurities;
	• High leaching efficiency;	• Low need for external reagents.		• Express REE-binders to couple leaching with selective biosorption.
	• REE tolerance.			
*Methylobacterium extorquens* AM1	• Using e-wastes as REE resources to enable methanol metabolism.	• Natural bioaccumulation of Ln^3+^ during methylotrophic growth.	• Has not been broadly engineered and studied.	• Enhance organic acid production;
	• Enhanced Nd bioaccumulation through *ppx* gene depletion.			• Co-deploy peptide/protein biosorption for higher REE accumulation.
*Gluconobacter oxidans* B58	• Knockout screens found 89 Nd-leach genes;	• Fast and highly acidic biolixiviant production (gluconic acid-rich);	• Unknown REE selectivity.	• Optimize aeration and S/L for improve yields
	• Engineered strains resulted in more acidic biolixiviants, lowering pH by 0.39 units, enhancing REE bioleaching by 53% at 10% w/V S/L ratio and by 73% at 1% w/V S/L ratio, respectively.	• Relatively clear REE metabolism.		• Express REE-binders to couple leaching with selective biosorption.
		• Acid tolerance		
*Methylacidiphilum fumariolicum SolV*	• Selectively uptake LREEs and HREEs by adjusting REE concentrations.	• Robust under minimal and extreme conditions, e.g., acidic pH, high temperatures, and the presence of toxic heavy metals like Th and U.	• Has not been broadly engineered and studied.	• Use multicycle accumulation;
				• Adjust feed REE concentrations to tune selectivity.
*Penicillium expansum*	• At an initial pH of 7.5, with 0.1 mM phosphate, substantial extraction of La (40%) and Tb (50%) was achieved, along with notable recovery of Pr, Nd, and Gd, reaching nearly 70% within 24 h.	• Fast leaching associated with the production of a gluconic acid-rich biolixiviant;	• Nonspecific: codissolution (e.g., Fe) hindering REE recovery.	• Express REE-binders to couple leaching with selective biosorption.
		• Comparable efficacy of using cell-free supernatant.		

### 4.1 Natural/Engineered Microorganisms for REE Biorecovery

#### 4.1.1 E. coli

*E. coli* has emerged as a versatile microbial chassis for REE recovery due to its well-characterized genetics, ease of manipulation, and potential for engineering bioleaching and biosorption functions, including organic acid secretion,^97,98^ REE-binding compounds expression,^55,64^ surface display technologies,^71,99^ and use as a whole-cell biosorbent.^72,77^ However, pH fluctuations in growth conditions pose a major stress for most neutralophilic bacteria, including *E. coli*, highlighting the need for future research to focus on uncovering acid-tolerance mechanisms in microorganisms and applying them to enhance *E. coli*’s resilience.^100^ Furthermore, the natural binding preference of unmodified *E. coli* cells for Yb and Lu suggests that it could serve as an effective biosorption platform when combined with other REE-binding substances targeting these elements, enabling more selective and efficient recovery.

#### 4.1.2 Acidithiobacillus

*Acidithiobacillus* species are acidophilic, chemolithoautotrophic bacteria widely studied for their role in metal biorecovery, particularly through bioleaching. They oxidize sulfur and iron compounds to generate sulfuric acid and ferric ions, which help solubilize REEs from ores and e-waste under highly acidic conditions. Their natural acid tolerance and metabolic versatility make them effective agents for sustainable and low-cost REE extraction from various solid matrices. Therefore, the mechanism of their high REE tolerance was investigated and revealed the significant role of their outer membrane and cell wall compared to *E. coli*.^101^ In addition, due to their high leaching efficiency, *Acidithiobacillus* species have been genetically engineered to express REE-binding proteins such as LanM, enabling the integration of bioleaching with selective REE biosorption for the development of advanced microbial platforms for REE recovery.^20^ However, the bioleaching process mediated by *Acidithiobacillus* is typically nonspecific, leading to the codissolution of other metals, which can hinder REE recovery from e-waste. Therefore, pretreatment steps, such as the removal of interfering metals like iron, are essential to enhance REE bioavailability and improve overall leaching efficiency.^102^

#### 4.1.3 *M. extorquens* AM1

*M. extorquens* AM1 is a well-characterized methylotrophic bacterium that utilizes methanol and other one-carbon compounds as its sole sources of carbon and energy.^103^ Its distinctive metabolic pathways make it a valuable tool for various applications, such as bioremediation and synthetic biology. Notably, in the realm of REEs, *M. extorquens* AM1 has drawn significant interest due to its reliance on lanthanides for the activity of MDH.^104^ The lanthanide-dependent XoxF-type MDH enables efficient methanol oxidation, offering key insights into REE biochemistry and presenting opportunities for their recycling or biomining. In addition, *M. extorquens* AM1 was previously reported to grow by using e-wastes as REE resources to enable methanol metabolism, especially with the addition of organic acids,^105^ and has been successfully engineered for the production of itaconic acid (ITA),^106^ a naturally occurring dicarboxylic acid produced by fungi such as *Aspergillus terreus*. ITA can solubilize REEs in solid matrices through chelation and acidic leaching, achieving pH and temperature-regulated selective leaching and precipitation,^22,107^ and easy downstream purification by peptide- and protein-based biosorption. Despite the significant attention it has received, few platforms utilizing *M. extorquens* AM1 for REE biorecovery have been developed.

#### 4.1.4 *G. oxidans* B58

*G. oxidans* B58 is considered one of the most promising microorganisms for REE bioleaching. This microorganism possesses a unique ability to secrete organic acids and generate a highly acidic biolixiviant, primarily composed of gluconic acid, which is especially effective for recovering REEs from various sources, including e-waste such as NiMH batteries.^50^ Sabrina et al. developed a highly nonredundant whole-genome knockout collection and screened this collection for Nd bioleaching from synthetic monazite.^108^ This work identified 89 important genes for bioleaching Nd and eight genetically modified *G. oxidans* strains with up to 111% increased REE extraction efficiency. Minimal pH changes in most cases suggested that nonacidic mechanisms play a key role. Alexa et al. generated a whole-genome knockout collection of single-gene transposon disruption mutants for *G. oxidans* B58.^109^ Among the 304 genes identified as influencing REE bioleaching, disruptions in key pathways, such as PQQ biosynthesis and glucose dehydrogenase, significantly impaired bioleaching performance, whereas mutations in phosphate transport genes enhanced extraction efficiency by up to 18%. Consequently, based on the genetic mechanisms identified for enhancing REE-bioleaching, researchers engineered *G. oxidans* B58 through a clean deletion of the phosphate transport gene *pstS* combined with the overexpression of the membrane-bound glucose dehydrogenase gene (*mgdh*) through the P112 promoter. These modifications resulted in more acidic biolixiviants, lowering pH by 0.39 units, enhancing REE bioleaching by 53% at 10% w/V S/L ratio and by 73% at 1% w/V S/L ratio, respectively.^39^

In addition to the above-mentioned strains, microbiomes such as *Methylacidiphilum fumariolicum* (*M. fumariolicum*) SolV and *Penicillium expansum* (*P. expansum*) have also been studied and optimized to enhance REE biorecovery efficiency from various wastes. Helena et al. demonstrate that strain *M. fumariolicum* SolV can grow robustly under minimal and extreme conditions, including acidic pH, high temperatures, and the presence of toxic heavy metals like Th and U, and can selectively uptake LREEs and HREEs by adjusting REE concentrations, highlighting the promise of leveraging naturally evolved bacterial systems for the selective and sustainable recovery of critical lanthanides.^110^ Alejandra et al. optimized REE bioleaching from WPCB using *P. expansum*, demonstrating high efficiency under optimal conditions, an initial pH of 7.5, 0.1 mM phosphate concentration, and no buffering agent.^111^ Under these conditions, the study achieved substantial extraction of La and Tb, as well as notable recovery of Pr, Nd, and Gd, reaching nearly 70% within 24 h. The bioleaching mechanism was attributed to the production of a gluconic acid-rich biolixiviant and the activity of the fungal plasma membrane proton pump, an essential enzyme that helps keep intracellular pH and membrane potential through actively pumping protons out of the cell using ATP.^112^ Moreover, the similar performance of cell-free supernatant and crude biolixiviant suggests a simplified and scalable approach, highlighting this method as a promising and sustainable biotechnology for REE recovery from e-waste.

Overall, *E. coli* is a versatile recombinant chassis that has been engineered to express surface-displayed peptides, protein binders, and other sorbents for REE capture, enabling efficient and partially selective recovery. However, fine discrimination among individual REEs remains challenging due to the scarcity of highly specific binders. Acid-tolerant, organic-acid–secreting microorganisms (e.g., *Acidithiobacillus*, *G. oxidans*, and *P. expansum*) are ideal platforms for optimizing bioleaching under harsh conditions and could be further engineered to incorporate high-affinity, REE-selective binding and uptake modules to improve end-to-end selectivity. For naturally REE-dependent strains (e.g., *M. extorquens* AM1), systems-level metabolic analyses (e.g., transcriptomics, proteomics, metabolomics) can elucidate essential REE-involved pathways, thereby guiding engineering strategies for more efficient and selective bioaccumulation.

### 4.2 Essential Parameters for Advanced REE Biorecovery

The microorganism-based biorecovery of REEs is an emerging green technology that leverages microbial processes to extract and concentrate REEs from e-waste. The efficiency and selectivity of these biological systems are governed by a range of essential physicochemical and biological parameters. One of the most critical parameters is pH, as it affects both microbial viability and metal solubility. For instance, acidophilic microbes like *Acidithiobacillus* thrive in low pH environments (pH ∼ 2) conducive to REE solubilization, while neutrophilic strains like *E. coli* require strategies to cope with acid stress. The initial pH and phosphate concentration of culture medium have also been reported as the determining factor of bioleaching and biosorption efficiency, as an initial pH of 7.5 for bioleaching by *P. expansum* and a pH of ∼ 5 for biosorption by *Galdieria sulphuraria*.^111,113^ In addition, most current studies employ a pH-regulated elution and biosorbent reuse methodology for downstream REE separation, allowing for gradient separation of REEs and the reuse of biosorbents.^61,114,115^ Therefore, optimizing microorganism-based REE biorecovery systems requires careful consideration of the initial pH of the culture medium and the concentration of preadded nutrients and buffering agents.

Redox potential (Eh) influences the oxidation state of metals and microbial metabolism, particularly in systems relying on iron or sulfur oxidation. In the case of *Acidithiobacillus*, high Eh values promote the oxidation of Fe^2+^ to Fe^3+^, which acts as a chemical oxidant to enhance REE leaching from minerals such as monazite.^116^ Temperature directly impacts microbial growth and enzymatic activity, with different strains showing optimal performance under mesophilic or thermophilic conditions. The choice of microbial species or engineered strains determines the specific mechanisms of REE interaction, including organic acid production, REE-binding protein expression (e.g., LanM), and cell surface modifications. Metal speciation and the presence of competing ions such as Fe^3+^ or Al^3+^ can inhibit REE recovery, necessitating pretreatment steps or the use of highly selective biosorbents.^102,117^ Additionally, biomass concentration (S/L ratio), contact time, and system design (batch vs continuous) influence the kinetics and scalability of recovery.^118^ A comprehensive understanding and optimization of these parameters is essential for the development of robust and efficient microbial platforms tailored for REE recovery from complex e-waste.

## 5 Conclusions and Outlook

Microorganism-based REE biorecovery presents a promising and sustainable alternative to conventional extraction methods, particularly for recovering valuable elements from e-waste. Organic acid-producing microbes such as *G. oxidans* and *P. expansum* have demonstrated efficient REE bioleaching through the secretion of gluconic acid-rich biolixiviants, with optimized culture conditions, such as moderate initial pH, low phosphate concentrations, and minimal buffering, greatly enhancing performance. Acidophilic bacteria like *Acidithiobacillus* further contribute to nonspecific but robust REE solubilization under low pH, though selectivity remains a challenge. To address this, biosorption and bioaccumulation strategies using a range of REE-binding substances have shown great promise. These include natural metalloproteins such as LanM, engineered peptides like lanthanide binding tags, and nucleic acid structures such as G-quadruplexes, which offer specific binding affinities toward LREEs or HREEs through well-defined coordination sites. *E. coli* has been widely explored as a synthetic biology chassis for incorporating these substances, enabling tailored and modular REE recovery systems. Recent genome-wide knockout studies in *G. oxidans* have also revealed previously unknown genes involved in REE bioleaching, many of which function through nonacidic pathways, such as membrane transport and metal homeostasis.

Looking ahead, the integration of bioleaching and biosorption mechanisms into unified microbial platforms, supported by genetic engineering, metabolic optimization, and well-controlled culture conditions (e.g., pH, nutrients, temperature, redox potential, and metal ion speciation), will be a key to advancing REE biorecovery (Figure 3).

**Figure 3:**
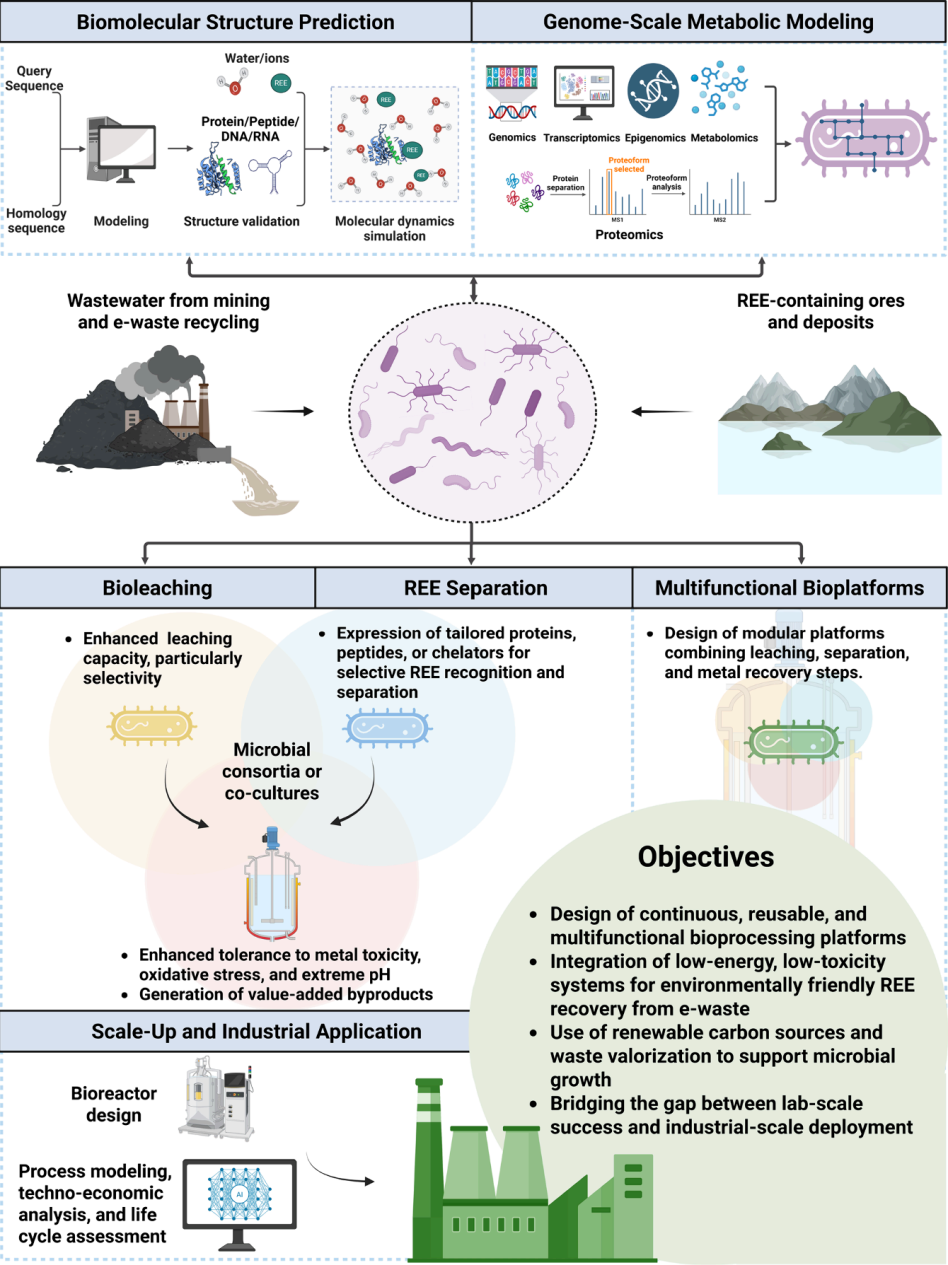
Integrated strategy for advancing microorganism-based REE biorecovery with enhanced efficiency, selectivity, robustness, and scalability.

Natural stains that are isolated from REE-containing ores/deposits, mining wastes, and e-waste recycling with potential REE leaching and uptake performance, strong tolerance to acidic and high-temperature conditions, and the capability to escape from metal toxicity could be first identified and analyzed by advanced molecular techniques, e.g., genomics, transcriptomics, epigenomics, metabolomics, and proteomics. Based on these data, genomic-scale metabolic modeling is able to profile the essential genes monitoring such REE involved metabolic activities, providing instructions in the optimization of metabolic pathways involved in REE mobilization and separation.^119^ Similarly, advances in biomolecular structure prediction, including homology modeling, structure validation, and molecular dynamics simulations, will enable the rational design of proteins, peptides, and chelators with enhanced REE-binding specificity.^120,121^ These data-driven approaches will broaden strain engineering methodologies and accelerate the targeted enhancement of microbial performance.

In terms of bioleaching, engineered microbial strains or consortia can be tailored to achieve greater leaching efficiency and improved selectivity for REEs over base metals. By enhancing tolerance to metal toxicity, oxidative stress, and extreme pH, these microorganisms can operate effectively under industrially relevant and often harsh conditions. Co-culturing strategies may also allow for synergistic activity, combining leaching and selective binding or separation within a single microbial community. These biological enhancements have the potential to reduce reliance on harsh chemical leaching agents while maintaining or improving recovery yields and selectivity. Beyond leaching, the targeted separation of REEs will be advanced through the expression of designed biomolecules such as REE-specific peptides and engineered metalloproteins. These molecules can provide high affinity and selectivity, enabling efficient capture of specific REEs from complex matrices of e-waste leachates. Integrating bioleaching and separation within modular, multifunctional platforms will reduce processing steps, improve recovery efficiency, and allow the reuse of microbial systems. Importantly, certain microorganisms can produce bioderived substances, such as phosphates, carbonates, or oxalates, that react with REEs to form insoluble mineral phases, providing a selective precipitation route for metal recovery.^122^ This biogenic precipitation can be coupled directly with bioleaching to create a closed-loop recovery process.^123,124^ In addition, microbial metabolism can yield value-added byproducts such as organic acids, biosurfactants, biopolymers, or pigments, which can be harvested alongside REEs to improve the economic viability of the process.^125–127^

From an industrial perspective, the scalability of microorganism-based REE recovery will rely on robust bioreactor designs and process optimization. Heterogeneity characterization (e.g., REEs and base metal composition) and suitable pretreatments of e-waste, such as delamination, liberation, contaminant removal, and oxidative or alkali treatments, are also essential to enhance the efficiency and selectivity of bioleaching and biosorption, particularly by exposing REE phases and mitigating interference from competing base metals.^45,128,129^ Continuous bioprocesses capable of supporting long-term microbial activity will be essential for high-throughput operations. A productive architecture is (i) continuous biolixiviant generation (e.g., organic acids) feeding (ii) controlled leaching contactors, followed by (iii) solid–liquid separation and (iv) continuous-flow biosorption, then (v) REEs elution and (vi) precipitation/roasting to REE oxides. Process modeling and techno-economic analysis (TEA) will guide the optimization of operating conditions, feedstock compatibility, energy use, and cost-effectiveness.^130^ Such developments will help bridge the gap between laboratory-scale success and industrial-scale deployment. Sustainability will remain a central driver of this field. Reducing environmental impacts in REE recycling can be achieved by integrating life cycle assessment (LCA) into biomining process development. LCA will enable a comprehensive evaluation of environmental burdens, from the sourcing of REE-containing waste materials, through bioleaching and separation, to final product recovery efficiency and residual waste management. By identifying hotspots such as high chemical usage, energy demand, or wastewater generation, LCA can provide actionable insights for optimizing microbial strains, refining process conditions, and improving resource efficiency. Employing renewable carbon sources and valorizing waste streams to support microbial growth will reduce environmental impact and contribute to circular economy goals. One TEA/LCA study for optimizing a bioleaching process using *G. oxidans* revealed that carbon/energy sources are the main economic bottleneck and the process can be marginally profitable, with profitability depending on cheaper carbon/energy sources, higher REE content in feedstock (>1.5% by mass), and improved leaching efficiency.^131^ TEA for integrating biosorption in REEs recovery also indicated that profitability depends on REE concentrations, feedstock compositions, and pretreatment/waste management costs, and biosorption is particularly viable for low-grade feedstocks, whereas high-grade resources may not be economically favorable.^132^ Moreover, low-energy, low-toxicity biobased methods for REE recovery will offer a greener alternative to conventional hydrometallurgical and pyrometallurgical processes. A TEA/LCA of an acid-free dissolution route for recovering didymium oxide from hard-drive shreds found that a plant processing 342.42 t HDD shreds/year would produce 2.53 t didymium oxide.^133^ With further optimization, the minimum selling price (MSP) could fall to ∼ $73/kg, and the greenhouse gas footprint was estimated at 4.91 kg CO_2_ per kg REE. Sensitivity analysis identified REE and CuSO_4_ recovery efficiencies and the HDD REE content as the main MSP drivers. Compared with hydrometallurgical and electrometallurgical options, the acid-free dissolution pathway appears particularly attractive for e-waste recovery. In the long term, the combination of synthetic biology, computational design, and scalable engineering promises to deliver resilient, selective, and multifunctional microbial platforms capable of closing the loop on REE recovery from secondary resources like e-waste and beyond (e.g., coal byproducts and mining wastewater).

## Abbreviations

REEs: Rare earth elements
LREEs: Light rare earth elements
HREEs: Heavy rare earth elements
e-waste: Electronic waste
WPCBs: Waste printed circuit boards
LanM: Lanmodulin
LanP: Lanpepsy
PQQ: Pyrroloquinoline quinone
PqqT: Pyrroloquinoline quinone-binding protein
NdFeB magnets: Neodymium magnets
NiMH batteries: Nickel metal-hydride batteries
HDDs: Hard disk drives
D2EHPA: Di­(2-ethylhexyl)­phosphoric acid
S/L: Solid-to-liquid
NADESs: Natural deep eutectic solvents
DESs: Deep eutectic solvents
EG-MA: Ethylene glycol-maleic acid
SELEX: Systematic evolution of ligands by exponential enrichment
MDH: Methanol dehydrogenase
ITA: Itaconic acid
TEA: Techno-economic analysis
LCA: Life cycle assessment
